# Supplementary material for: Role and mechanism of NCAPD3 in promoting malignant behaviors in gastric cancer
Source: Front Pharmacol. 2024 Apr 22;15:1341039. doi: 10.3389/fphar.2024.1341039 (PMC11070777; doi:10.3389/fphar.2024.1341039)
Supplement: Supplementary file 11 [file DataSheet2.ZIP › GSEA/immunologic/my_analysis.Gsea.1599462003817/GSE17974_0H_VS_24H_IN_VITRO_ACT_CD4_TCELL_DN.html]

Details for gene set GSE17974\_0H\_VS\_24H\_IN\_VITRO\_ACT\_CD4\_TCELL\_DN[GSEA]

|  || Dataset | filtered\_dataset.sample\_info.cls#WT\_versus\_NCAPD3\_MUT |
| Phenotype | sample\_info.cls#WT\_versus\_NCAPD3\_MUT |
| Upregulated in class | WT |
| GeneSet | GSE17974\_0H\_VS\_24H\_IN\_VITRO\_ACT\_CD4\_TCELL\_DN |
| Enrichment Score (ES) | 0.438844 |
| Normalized Enrichment Score (NES) | 2.0124545 |
| Nominal p-value | 0.0016286644 |
| FDR q-value | 0.5650311 |
| FWER p-Value | 0.997 |
Table: GSEA Results Summary

  

Fig 1: Enrichment plot: GSE17974\_0H\_VS\_24H\_IN\_VITRO\_ACT\_CD4\_TCELL\_DN      
 Profile of the Running ES Score & Positions of GeneSet Members on the Rank Ordered List

  

| SYMBOL | TITLE | RANK IN GENE LIST | RANK METRIC SCORE | RUNNING ES | CORE ENRICHMENT || 1 | 8568 | RRP1 | 22 | 1.027 | 0.0570 | Yes |
| 2 | 80746 | TSEN2 | 53 | 0.909 | 0.0998 | Yes |
| 3 | 27304 | MOCS3 | 76 | 0.844 | 0.1438 | Yes |
| 4 | 284323 | ZNF780A | 90 | 0.805 | 0.1916 | Yes |
| 5 | 23450 | SF3B3 | 178 | 0.697 | 0.1783 | Yes |
| 6 | 9456 | HOMER1 | 198 | 0.680 | 0.2128 | Yes |
| 7 | 55028 | C17orf80 | 222 | 0.659 | 0.2430 | Yes |
| 8 | 6573 | SLC19A1 | 231 | 0.650 | 0.2833 | Yes |
| 9 | 55711 | FAR2 | 262 | 0.622 | 0.3058 | Yes |
| 10 | 25974 | MMACHC | 265 | 0.621 | 0.3485 | Yes |
| 11 | 25973 | PARS2 | 266 | 0.620 | 0.3925 | Yes |
| 12 | 63920 | ZBED8 | 298 | 0.593 | 0.4122 | Yes |
| 13 | 197259 | MLKL | 346 | 0.569 | 0.4187 | Yes |
| 14 | 8187 | ZNF239 | 440 | 0.508 | 0.3876 | Yes |
| 15 | 29071 | C1GALT1C1 | 457 | 0.500 | 0.4115 | Yes |
| 16 | 79080 | CCDC86 | 555 | 0.443 | 0.3730 | Yes |
| 17 | 5198 | PFAS | 570 | 0.437 | 0.3939 | Yes |
| 18 | 23404 | EXOSC2 | 609 | 0.418 | 0.3962 | Yes |
| 19 | 54512 | EXOSC4 | 623 | 0.411 | 0.4159 | Yes |
| 20 | 51728 | POLR3K | 646 | 0.401 | 0.4285 | Yes |
| 21 | 6182 | MRPL12 | 729 | 0.359 | 0.3948 | Yes |
| 22 | 55759 | WDR12 | 731 | 0.359 | 0.4196 | Yes |
| 23 | 55646 | LYAR | 761 | 0.342 | 0.4229 | Yes |
| 24 | 51118 | UTP11L | 773 | 0.336 | 0.4388 | Yes |
| 25 | 6385 | SDC4 | 871 | -0.282 | 0.3889 | No |
Table: GSEA details [plain text format]

  

Fig 2: GSE17974\_0H\_VS\_24H\_IN\_VITRO\_ACT\_CD4\_TCELL\_DN      
 Blue-Pink O' Gram in the Space of the Analyzed GeneSet

  

Fig 3: GSE17974\_0H\_VS\_24H\_IN\_VITRO\_ACT\_CD4\_TCELL\_DN: Random ES distribution      
 Gene set null distribution of ES for **GSE17974\_0H\_VS\_24H\_IN\_VITRO\_ACT\_CD4\_TCELL\_DN**
